# Vitamin D deficiency and risk of acute kidney injury after ischemic stroke: a propensity-matched cohort study

**DOI:** 10.3389/fnut.2025.1709491

**Published:** 2026-01-05

**Authors:** Kuo-Chuan Hung, Li-Chen Chang, Yi-Chen Lai, Ming Yew, Ping-Hsin Liu, I-Wen Chen

**Affiliations:** 1 Department of Anesthesiology, Chi Mei Medical Center, Tainan, Taiwan; 2 School of Medicine, College of Medicine, National Sun Yat-sen University, Kaohsiung, Taiwan; 3 Department of Anesthesiology, E-Da Hospital, I-Shou University, Kaohsiung, Taiwan; 4 Department of Anesthesiology, Chi Mei Medical Center, Liouying, Tainan, Taiwan

**Keywords:** vitamin D deficiency, acute kidney injury, ischemic stroke, retrospective study, propensity score matching

## Abstract

**Background:**

Vitamin D deficiency (VDD) has been linked to adverse outcomes in various clinical settings, but its relationship with post-stroke acute kidney injury (AKI) remains unexplored.

**Methods:**

This retrospective cohort study utilized the TriNetX research network database to identify adult patients with first-documented ischemic stroke between January 2010 and December 2024. Patients were stratified based on serum 25-hydroxyvitamin D levels measured within 6 weeks pre-stroke: VDD group (<20 ng/mL) and control group (≥30 ng/mL). After 1:1 propensity score matching, we compared 30-day and 1–12 month outcomes between groups, with the primary outcome being AKI incidence within 30 days post-stroke.

**Results:**

After matching (*n* = 4,343 per group), patients with pre-stroke VDD demonstrated significantly higher 30-day AKI incidence compared with those with sufficient vitamin D levels (5.3% vs. 3.5%; odds ratio [OR] = 1.55, 95% confidence interval [CI] 1.26–1.92; *p* < 0.001). VDD was also associated with increased risks of all-cause mortality (OR = 1.63, 95% CI 1.26–2.12), intensive care unit (ICU) admission (OR = 1.55, 95% CI 1.30–1.84), pneumonia (OR = 1.48, 95% CI 1.09–2.02), and dialysis requirement (OR = 2.33, 95% CI 1.36–4.00). Vitamin D insufficiency (20–29 ng/mL) was associated with a milder but significant AKI risk increase (OR = 1.39, 95% CI 1.12–1.72; *p* = 0.003). The adverse effect of VDD persisted during 1–12 month follow-up, with higher risks of AKI (hazard ratio [HR] = 1.32, 95% CI 1.13–1.55) and progression to end-stage renal disease (HR = 1.69, 95% CI 1.16–2.46).

**Conclusion:**

Pre-stroke VDD is associated with increased risk of post-stroke AKI and other adverse outcomes. The observed dose-dependent relationship suggests potential benefits from optimizing vitamin D status. These findings highlight the importance of assessing vitamin D levels in stroke risk stratification and suggest potential preventive strategies.

## 1 Introduction

Globally, stroke continues to be a primary cause of death and disability (1). The global burden of disease data from 2019 documented 12.2 million new stroke cases, with ischemic strokes constituting the majority (62.4%) (2). Acute kidney injury (AKI) frequently complicates stroke recovery, occurring in 11.6–20.9% of patients with post-stroke (3–7). The clinical impact of this complication is substantial, patients developing AKI after stroke experience 2–3 times higher in-hospital mortality rates (7–9), longer hospitalizations, and greater risk for developing chronic kidney disease (10, 11). A particularly concerning finding is the association between post-stroke AKI and poorer functional outcomes; research shows that 88.1% of stroke patients with AKI demonstrate moderate to severe disability at 3-month follow-up, compared to 51.3% of those without AKI (12). These impairments typically persist, necessitating extended rehabilitation and diminishing quality of life. Identifying modifiable risk factors for post-stroke AKI is crucial for developing effective preventive strategies that could improve recovery trajectories and long-term outcomes.

Vitamin D deficiency (VDD) has been increasingly recognized as a potential risk factor for various adverse outcomes across different clinical settings (13–16). While previous research has established relationships between VDD and increased stroke risk (17–19) as well as between VDD and AKI in other patient populations (20–22), the specific association between pre-stroke vitamin D status and post-stroke AKI remains inadequately explored. Moreover, evidence from prospective cohort studies indicates that the strength of association between baseline serum 25-hydroxyvitamin D and stroke risk may attenuate with longer follow-up durations, as participants’ vitamin D status can change over time, potentially diluting the observed relationship (23). Biological plausibility for such an association exists, as vitamin D has known renoprotective properties through multiple mechanisms, including regulation of the renin-angiotensin-aldosterone system, anti-inflammatory effects, maintenance of endothelial function, and mitigation of oxidative stress (24–29), all of which may be particularly relevant in the post-stroke setting where hemodynamic instability and systemic inflammation can compromise renal function (30, 31). Recent reviews further support these mechanisms, emphasizing the renoprotective actions of vitamin D and its receptor activators in diabetic and chronic kidney disease through anti-proteinuric, anti-fibrotic, and anti-inflammatory pathways (32–34).

Given these observations across diverse clinical scenarios and the biological mechanisms involved, we hypothesized that pre-stroke VDD was associated with increased risk of post-stroke AKI. This study aimed to examine the relationship between pre-stroke vitamin D status and the risk of developing AKI after ischemic stroke during both short-term (30-day) and long-term (1–12 month) follow-up periods.

## 2 Methods

### 2.1 Data source and ethical statement

This retrospective, multi-center cohort study was conducted using data obtained from the TriNetX research network, a global health research platform that aggregates de-identified electronic medical records from participating healthcare organizations, predominantly located in the United States. The TriNetX database offers access to a large-scale, real-world dataset encompassing a wide range of clinical variables, including patient demographics, diagnoses (ICD codes), procedural and medication histories, laboratory results, and longitudinal clinical outcomes. The study protocol was reviewed and approved by the Institutional Review Board (IRB) of Chi Mei medical center (IRB number 11310-E04). Given that all data used in the analysis were de-identified and posed minimal risk to patient privacy, the IRB granted a waiver of informed consent in accordance with applicable ethical guidelines.

### 2.2 Patient selection and inclusion criteria

We carried out a retrospective cohort study to find adult patients (aged 18 and above) who had their first recorded ischemic stroke between January 2010 and December 2024. Ischemic strokes were identified using the ICD-10-CM code I63, which includes both acute and subacute cases. Patients were stratified into two cohorts based on their serum 25-hydroxyvitamin D [25(OH)D] levels measured within 6 weeks prior to the stroke event: the VDD group, defined as having 25(OH)D < 20 ng/mL, and the control group, defined as having 25(OH)D ≥ 30 ng/mL. In current study, the term “control group” refers to patients with sufficient vitamin D levels, serving as a comparison cohort rather than a randomized control arm. To ensure accurate classification, individuals in the VDD group were excluded if any 25(OH)D measurement during the defined period exceeded 20 ng/mL. Similarly, patients in the control group were excluded if any value fell below 30 ng/mL. Our selection of a six-week window for assessing pre-stroke vitamin D levels was based on prior observational studies indicating that serum 25(OH)D levels remain relatively stable over short intervals and reflect recent vitamin D status.

Additional exclusion criteria were: (1) intracerebral hemorrhage within 1 year before the stroke; (2) history of pneumonia, intensive care unit (ICU) admission, COVID-19 infection, AKI, or intracranial injury within 3 months before the stroke; (3) history of cystic kidney disease, end-stage renal disease (ESRD), or systemic lupus erythematosus (SLE); (4) surgery or bleeding event within 2 weeks before the stroke; and (5) contrast-induced AKI within 2 weeks after the stroke.

### 2.3 Data collection and propensity score matching

We extracted baseline demographic (e.g., age and body mass index), preexisting comorbidities (e.g., hypertension, diabetes mellitus, ischemic heart disease, neoplasms, cerebrovascular disease), relevant laboratory values (e.g., hemoglobin, serum albumin), and outpatient medication usage (e.g., angiotensin-converting enzyme (ACE) inhibitors and angiotensin II receptor blockers) from the TriNetX research network. A detailed summary of all variables included in the analysis is presented in Table 1.

**Table 1:** Baseline characteristics of patients with ischemic stroke before and after propensity score matching.

Variables	Before matching		After matching			
	VDD group (*n* = 4,932)	Control group (*n* = 9,644)	SMD†	VDD group (*n* = 4,343)	Control group (*n* = 4,343)	SMD†
Patient characteristics						
Age at index (years)	63.7 ± 15.9	71.3 ± 13.3	0.521	65.9 ± 14.7	66.4 ± 14.9	0.029
BMI ≥ 30 (kg/m^2^)	1,199 (24.3%)	2077 (21.5%)	0.066	1,013 (23.3%)	984 (22.7%)	0.016
Female	2,665 (54.0%)	5,863 (60.8%)	0.137	2,400 (55.3%)	2,389 (55.0%)	0.005
White	2,611 (52.9%)	6,751 (70.0%)	0.356	2,536 (58.4%)	2,541 (58.5%)	0.002
Black or African American	1,213 (24.6%)	1,187 (12.3%)	0.321	845 (19.5%)	873 (20.1%)	0.016
Unknown Race	729 (14.8%)	1,020 (10.6%)	0.127	634 (14.6%)	626 (14.4%)	0.005
Factors influencing health status and contact with health services	2,438 (49.4%)	5,970 (61.9%)	0.253	2,222 (51.2%)	2,150 (49.5%)	0.033
Comorbidities/medication						
Essential (primary) hypertension	1947 (39.5%)	4,763 (49.4%)	0.200	1768 (40.7%)	1750 (40.3%)	0.008
Diabetes mellitus	1,158 (23.5%)	2,270 (23.5%)	0.001	1,018 (23.4%)	987 (22.7%)	0.017
Ischemic heart diseases	686 (13.9%)	1717 (17.8%)	0.107	637 (14.7%)	632 (14.6%)	0.003
Neoplasms	661 (13.4%)	2024 (21.0%)	0.202	626 (14.4%)	605 (13.9%)	0.014
Cerebrovascular diseases	637 (12.9%)	1,584 (16.4%)	0.099	586 (13.5%)	531 (12.2%)	0.038
Overweight and obesity	538 (10.9%)	959 (9.9%)	0.032	460 (10.6%)	449 (10.3%)	0.008
Heart failure	426 (8.6%)	890 (9.2%)	0.021	370 (8.5%)	359 (8.3%)	0.009
Nicotine dependence	455 (9.2%)	608 (6.3%)	0.109	365 (8.4%)	356 (8.2%)	0.008
COPD	297 (6.0%)	711 (7.4%)	0.054	278 (6.4%)	275 (6.3%)	0.003
Diseases of liver	240 (4.9%)	477 (4.9%)	0.004	213 (4.9%)	200 (4.6%)	0.014
COVID-19	66 (1.3%)	259 (2.7%)	0.096	65 (1.5%)	64 (1.5%)	0.002
Alcohol related disorders	118 (2.4%)	174 (1.8%)	0.041	104 (2.4%)	96 (2.2%)	0.012
Malnutrition	115 (2.3%)	207 (2.1%)	0.013	104 (2.4%)	109 (2.5%)	0.007
Chronic kidney disease, stage 1	15 (0.3%)	28 (0.3%)	0.003	12 (0.3%)	11 (0.3%)	0.004
Chronic kidney disease, stage 2	61 (1.2%)	165 (1.7%)	0.039	53 (1.2%)	56 (1.3%)	0.006
Chronic kidney disease, stage 3	316 (6.4%)	1,147 (11.9%)	0.191	303 (7.0%)	303 (7.0%)	<0.001
Chronic kidney disease, stage 4	93 (1.9%)	286 (3.0%)	0.070	88 (2.0%)	82 (1.9%)	0.010
Chronic kidney disease, stage 5	26 (0.5%)	45 (0.5%)	0.009	24 (0.6%)	22 (0.5%)	0.006
Laboratory data						
Hemoglobin ≥12 g/dL	2,743 (55.6%)	6,131 (63.6%)	0.163	2,484 (57.2%)	2,424 (55.8%)	0.028
Albumin g/dL (≥3.5 g/dL)	2,523 (51.2%)	6,300 (65.3%)	0.290	2,344 (54.0%)	2,270 (52.3%)	0.034
Hemoglobin A1c (>7%)	805 (16.3%)	1,181 (12.2%)	0.117	667 (15.4%)	658 (15.2%)	0.006
Glomerular filtration rate mL/min/1.73 m^2^	75.6 ± 28.4	67.8 ± 22.9	0.304	73.9 ± 27.3	72.9 ± 25.2	0.040
Medications						
ACE inhibitors	935 (19.0%)	1828 (19.0%)	<0.001	816 (18.8%)	801 (18.4%)	0.009
Angiotensin II inhibitor	572 (11.6%)	1,581 (16.4%)	0.139	534 (12.3%)	535 (12.3%)	0.001

BMI: body mass index; SMD: standardized mean differences; COPD: chronic obstructive pulmonary disease; †SMD values <0.1 indicate adequate balance between groups.

To reduce potential confounding and improve comparability between the VDD and control groups, we employed 1:1 propensity score matching (PSM) using a greedy nearest-neighbor algorithm with a caliper width of 0.1 pooled standard deviations. The propensity score model incorporated all variables listed in Table 1 to account for baseline differences. After matching, covariate balance between groups was evaluated using standardized mean differences (SMDs). Additionally, to visually assess the distributional overlap between the two groups, we generated propensity score density plots illustrating cohort comparability before and after matching.

### 2.4 Outcomes

The primary outcome of this study was the incidence of AKI within 30 days following the index ischemic stroke. Secondary outcomes included all-cause mortality, pneumonia, ICU admission, and the initiation of dialysis during the same 30-day post-stroke period. All outcomes were measured from the date of the index ischemic stroke to ensure consistency in follow-up timing. To further explore the potential long-term impact of VDD, we extended the analysis to include the same set of outcomes during the period from 30 to 365 days post-stroke. This dual time-frame approach allowed for evaluation of both short-term and longer-term clinical consequences potentially associated with pre-stroke VDD status.

### 2.5 Sensitivity analysis

To assess the robustness of our findings, three sensitivity analyses were performed. First, we restricted the analysis to patients who received care at medical centers (Model I), as these institutions are more likely to implement standardized protocols and provide advanced levels of care. This analysis aimed to evaluate whether higher-quality care settings could attenuate the association between VDD and adverse post-stroke outcomes by reducing variability in clinical management. Second, we limited the cohort to patients who experienced ischemic stroke between 2010 and 2019 (Model II). This time window was chosen to eliminate potential confounding introduced by the COVID-19 pandemic, which significantly disrupted healthcare delivery systems and clinical workflows beginning in 2020. Third, we conducted an analysis restricted to patients aged over 50 years (Model III) to account for potential age-related differences in baseline risk profiles and susceptibility to complications following stroke.

### 2.6 Analysis of vitamin D insufficiency and 30-day outcomes

To further investigate the relationship between suboptimal vitamin D levels and post-stroke complications, we conducted an additional analysis focusing on vitamin D insufficiency. Patients with serum 25(OH)D levels between 20 and 29 ng/mL measured within 6 weeks prior to the index ischemic stroke were identified as the insufficiency group. These patients were compared to those with sufficient vitamin D levels (25(OH)D ≥ 30 ng/mL) using 1:1 propensity score matching based on baseline demographic characteristics, comorbidities, laboratory parameters, and medication use (as detailed in Table 1).

### 2.7 Risk factors for AKI in patients with VDD

To identify specific risk factors associated with AKI development among patients with VDD, we performed multivariable logistic regression analysis. Variables included in the model were demographic characteristics (age, sex) and comorbidities (hypertension, diabetes mellitus, chronic kidney disease, heart failure, etc.). Adjusted odds ratios (aORs) with 95% confidence intervals (CIs) were calculated to quantify the strength of associations between potential risk factors and AKI development in this high-risk population.

### 2.8 Analysis of long-term outcomes

For the analysis of outcomes during the 1–12 month period following stroke, we employed time-to-event methods, calculating hazard ratios (HRs) with 95% CIs using Cox proportional hazards models. This approach allowed us to account for variable follow-up times during this extended observation period while comparing the rates of adverse outcomes between the VDD and control groups.

### 2.9 Statistical analysis

All statistical analyses were performed using TrinetX platform. Continuous variables were presented as mean ± standard deviation, while categorical variables were expressed as frequencies and percentages. Baseline characteristics between groups were compared using SMDs, with values <0.1 indicating adequate balance after propensity score matching. For 30-day outcomes, odds ratios (ORs) with 95% confidence intervals (CIs) were calculated to quantify effect sizes. For 1–12 month outcomes, Kaplan–Meier survival analysis and Cox proportional hazards models were used to estimate hazard ratios (HRs) with 95% CIs. Risk factors for AKI in patients with VDD were identified using multivariable logistic regression models, with results presented as adjusted odds ratios (aORs) with 95% CIs.

## 3 Results

### 3.1 Patient selection and baseline patient characteristics

**Figure 1:**
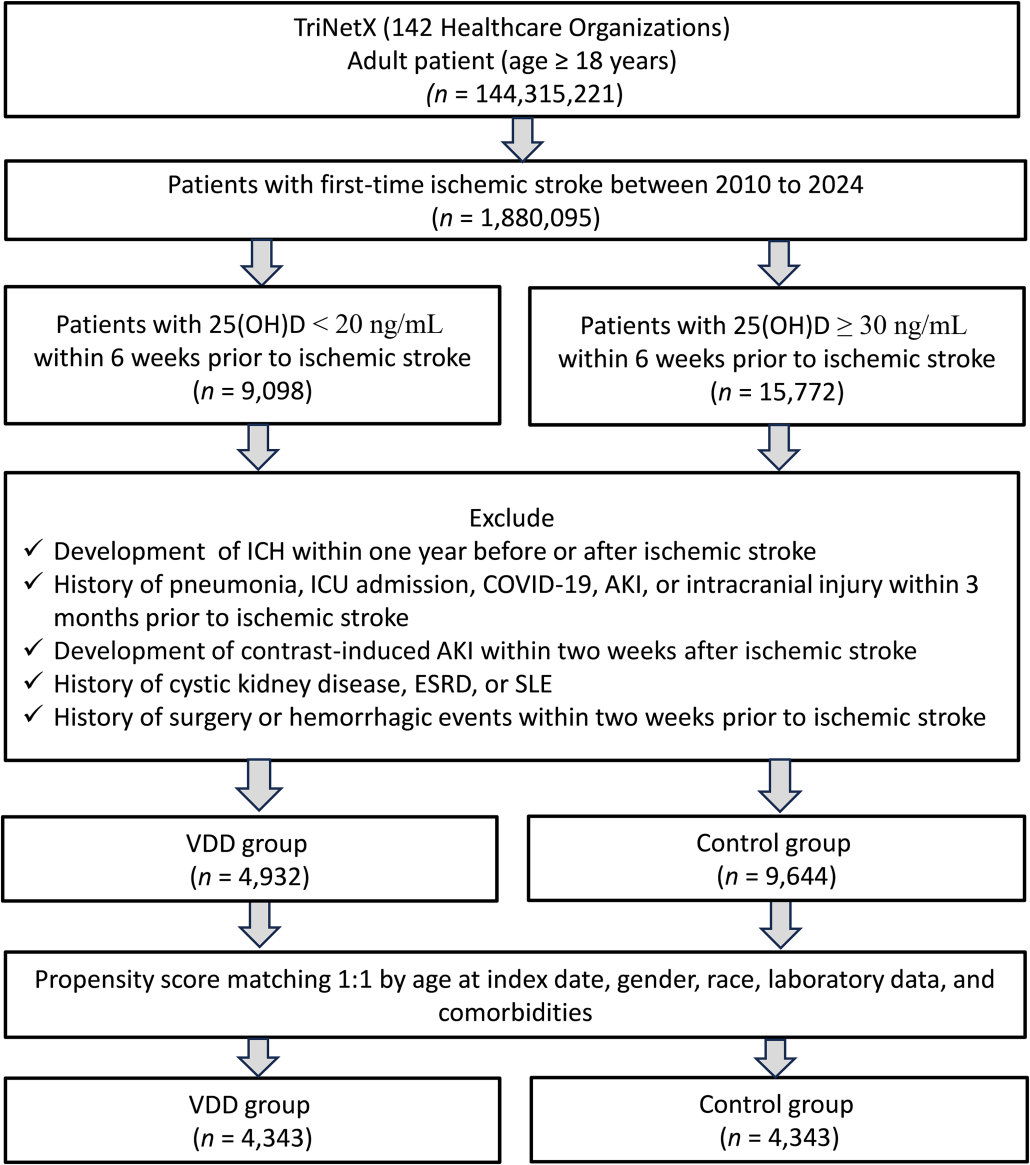
Patient selection from the TriNetX databases. AIS – Acute Ischemic Stroke; 25(OH)D – 25-hydroxyvitamin D; ICH – Intracerebral Hemorrhage; ICU – Intensive Care Unit; COVID-19 – Coronavirus Disease 2019; AKI – Acute Kidney Injury; ESRD – End-Stage Renal Disease; SLE – Systemic Lupus Erythematosus; VDD – Vitamin D Deficiency.

**Figure 2:**
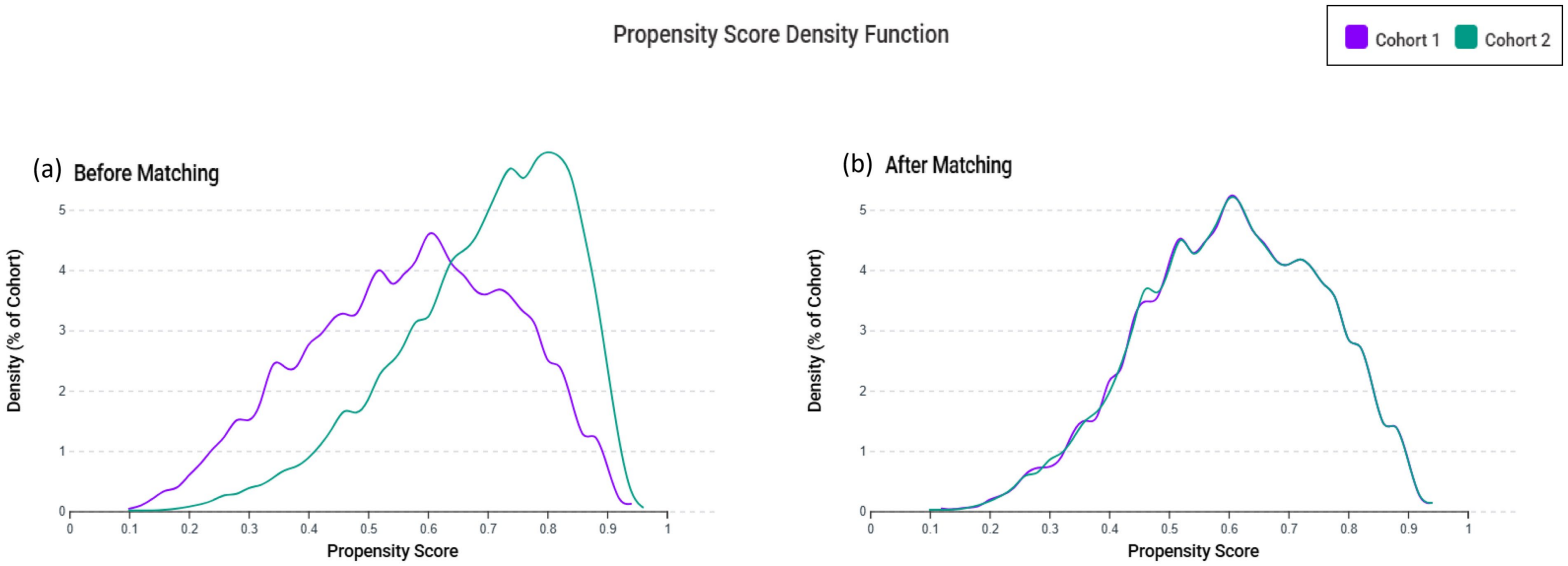
Propensity score distribution before and after matching. **(a)** Propensity score distribution before matching and **(b)** after matching between the vitamin D deficiency (Cohort 1) and control (Cohort 2) groups. The figure illustrates improved overlap following 1:1 propensity score matching, indicating adequate covariate balance across cohorts.

As illustrated in Figure 1, we initially identified patients with ischemic stroke from the TriNetX database. After applying our inclusion and exclusion criteria, a total of 4,932 patients with VDD and 9,644 patients with sufficient vitamin D levels (control group) were eligible for the study. Following 1:1 propensity score matching, we obtained two well-balanced cohorts, each comprising 4,343 patients. Table 1 presents the baseline characteristics of patients before and after propensity score matching. Prior to matching, several significant differences existed between the VDD and control groups. Patients in the VDD group were younger (63.7 ± 15.9 vs. 71.3 ± 13.3 years, SMD = 0.521), had a higher proportion of Black or African American individuals (24.6% vs. 12.3%, SMD = 0.321), and a lower proportion of White individuals (52.9% vs. 70.0%, SMD = 0.356). After matching, these differences were successfully eliminated, with all SMDs falling below 0.1, indicating well-balanced cohorts. The matched cohorts showed comparable distributions of comorbidities, such as hypertension (40.7% vs. 40.3%), diabetes mellitus (23.4% vs. 22.7%), and ischemic heart disease (14.7% vs. 14.6%). Laboratory parameters and medication usage were also similar between the matched groups. Figure 2 demonstrates the overlap of propensity score distributions before and after matching, confirming satisfactory balance between the VDD and control cohorts.

### 3.2 Association between pre-stroke VDD and 30-day outcomes

As shown in Table 2, patients with pre-stroke VDD demonstrated significantly higher rates of adverse 30-day outcomes compared to those with sufficient vitamin D levels. AKI occurred more frequently in the VDD group compared to the control group (5.3% vs. 3.5%, OR = 1.55, 95% CI: 1.26–1.92, *p* < 0.001). Similarly, patients with VDD experienced higher risks of all-cause mortality (OR = 1.63, 95% CI: 1.26–2.12, *p* < 0.001), ICU admission (OR = 1.55, 95% CI: 1.30–1.84, *p* < 0.001), and pneumonia (OR = 1.48, 95% CI: 1.09–2.02, *p* = 0.013). Notably, the requirement for dialysis was significantly more common in the VDD group (1.0% vs. 0.4%, OR = 2.33, 95% CI: 1.36–4.00, *p* = 0.002).

**Table 2:** Association between vitamin D deficiency (VDD) and 30-day outcomes.

Outcomes	VDD group (*n* = 4,343)	Control group (*n* = 4,343)	OR (95% CI)	*p*-value
	Events (%)	Events (%)		
Acute kidney injury	230 (5.3%)	151 (3.5%)	1.55 (1.26–1.92)	<0.001
All-cause mortality	153 (3.5%)	95 (2.2%)	1.63 (1.26–2.12)	<0.001
ICU admission	3,440 (7.8%)	226 (5.2%)	1.55 (1.30–1.84)	<0.001
Pneumonia	100 (2.3%)	68 (1.6%)	1.48 (1.09–2.02)	0.013
Dialysis	44 (1.0%)	19 (0.4%)	2.33 (1.36–4.00)	0.002

ICU: intensive care unit; ESRD: End-Stage Renal Disease; OR: odds ratio; CI: confidence interval.

### 3.3 Sensitivity analyses of the association between pre-stroke VDD and 30-day outcomes

To assess the robustness of our findings, we conducted three sensitivity analyses (Table 3). In Model I, which included only patients with stroke treated at medical centers (*n* = 3,280 per group), the association between VDD and AKI remained significant (OR = 1.47, 95% CI: 1.15–1.86, *p* = 0.002), although slightly attenuated compared to the main analysis. Model II, which restricted the analysis to patients with stroke between 2010 and 2019 (*n* = 2,521 per group), showed a stronger association between VDD and AKI (OR = 1.70, 95% CI: 1.26–2.29, *p* < 0.001) compared to the primary analysis. Similarly, Model III, which included only patients aged >50 years (*n* = 4,188 per group), confirmed the significant association between VDD and AKI (OR = 1.51, 95% CI: 1.23–1.87, *p* < 0.001).

**Table 3:** Sensitivity analyses of the association between vitamin D deficiency (VDD) and 30-day outcomes.

Outcomes	Model I		Model II		Model III	
	OR (95% CI)	*p*-values	OR (95% CI)	*p*-values	OR (95% CI)	*p*-values
Acute kidney injury	1.47 (1.15–1.86)	0.002	1.70 (1.26–2.29)	<0.001	1.51 (1.23–1.87)	<0.001
All-cause mortality	1.79 (1.32–2.43)	<0.001	2.14 (1.47–3.10)	<0.001	1.83 (1.40–2.38)	<0.001
ICU admission	1.55 (1.26–1.90)	<0.001	1.57 (1.25–1.97)	<0.001	1.58 (1.31–1.89)	<0.001
Pneumonia	1.69 (1.15–2.47)	0.006	1.45 (0.97–2.17)	0.069	1.77 (1.26–2.49)	<0.001
Dialysis	1.74 (0.92–3.29)	0.085	1.59 (0.77–3.28)	0.207	1.75 (1.04–2.92)	0.032

ICU: intensive care unit; OR: odds ratio; CI: confidence interval. Model I: Analysis of patients with stroke treated at medical center (*n* = 3,280 per group). Model II: Analysis of patients with stroke between 2010 and 2019 (*n* = 2,521 per group). Model III: Analysis of patients with age > 50 years (*n* = 4,188 per group).

The associations between VDD and mortality and ICU admission remained statistically significant across all three sensitivity models, with risks consistent with or greater than those observed in the main analysis. For pneumonia, the association remained significant in Model I (OR = 1.69, 95% CI: 1.15–2.47, *p* = 0.006) and Model III (OR = 1.77, 95% CI: 1.26–2.49, *p* < 0.001), but was not statistically significant in Model II (OR = 1.45, 95% CI: 0.97–2.17, *p* = 0.069). The association between VDD and dialysis varied across models. While the main analysis showed a significant association (OR = 2.33, *p* = 0.002), this finding was not consistently replicated in Model I (OR = 1.74, *p* = 0.085) and Model II (OR = 1.59, *p* = 0.207), though it remained significant in Model III (OR = 1.75, *p* = 0.032).

### 3.4 Analysis of vitamin D insufficiency and 30-day outcomes

We further investigated whether vitamin D insufficiency [serum 25(OH)D levels between 20 and 29 ng/mL] was associated with adverse 30-day outcomes compared to vitamin D sufficiency (≥30 ng/mL). As shown in Table 4, after matching (*n* = 4,414 per group), patients with vitamin D insufficiency demonstrated a significantly higher risk of AKI compared to those with sufficient vitamin D levels (4.7% vs. 3.4%, OR = 1.39, 95% CI: 1.12–1.72, *p* = 0.003). However, unlike the findings in the VDD group, vitamin D insufficiency was not significantly associated with increased risks of all-cause mortality, ICU admission, pneumonia, or dialysis requirement.

**Table 4:** Association between vitamin D insufficiency (VDI) and 30-day outcomes.

Outcomes	VDI group (*n* = 4,414)	Control group (*n* = 4,414)	OR (95% CI)	*p*-value
	Events (%)	Events (%)		
Acute kidney injury	208 (4.7%)	152 (3.4%)	1.39 (1.12–1.72)	0.003
All-cause mortality	105 (2.4%)	93 (2.1%)	1.13 (0.85–1.50)	0.388
ICU admission	251 (5.7%)	211 (4.8%)	1.20 (1.00–1.45)	0.056
Pneumonia	75 (1.7%)	75 (1.7%)	1.00 (0.72–1.38)	1.000
Dialysis	15 (0.3%)	19 (0.4%)	0.79 (0.40–1.55)	0.492

ICU: intensive care unit; OR: odds ratio; CI: confidence interval.

### 3.5 Risk factors for AKI at 30-day follow-up in patients with VDD

We identified several independent risk factors for AKI among patients with VDD (Table 5). After adjusting for potential confounders, chronic kidney disease emerged as a significant risk factor for AKI (adjusted OR = 1.95, 95% CI: 1.36–2.79, *p* < 0.001). Heart failure (adjusted OR = 1.97, 95% CI: 1.35–2.89, *p* < 0.001) and chronic obstructive pulmonary disease (adjusted OR = 1.84, 95% CI: 1.17–2.90, *p* = 0.008) were also associated with increased AKI risk. Additionally, older age (adjusted OR = 1.01 per year, 95% CI: 1.01–1.02, *p* = 0.002) and male sex (females had lower risk with adjusted OR = 0.56, 95% CI: 0.43–0.73, *p* < 0.001) were significant demographic risk factors for AKI development.

**Table 5:** Risk factors for acute kidney injury in patients with vitamin D deficiency.

Covariate	Crude OR (95% CI)	*P*-value	aOR (95% CI)	*P*-value
Age at Index	1.02 (1.01, 1.03)	<0.001	1.01 (1.01, 1.02)	0.002
Female	0.56 (0.44, 0.72)	<0.001	0.56 (0.43, 0.73)	<0.001
White	1.04 (0.81, 1.33)	0.770	0.93 (0.72, 1.20)	0.574
Essential (primary) hypertension	1.27 (0.99, 1.64)	0.059	1.02 (0.75, 1.38)	0.914
Overweight and obesity	1.29 (0.90, 1.86)	0.166	1.12 (0.74, 1.70)	0.592
Diabetes mellitus	1.34 (1.00, 1.79)	0.047	0.93 (0.66, 1.31)	0.667
Nicotine dependence	1.11 (0.73, 1.68)	0.639	0.74 (0.45, 1.21)	0.228
Ischemic heart diseases	1.84 (1.36, 2.50)	<0.001	0.97 (0.67, 1.42)	0.881
Chronic kidney disease (CKD)	2.68 (1.96, 3.65)	<0.001	1.95 (1.36, 2.79)	<0.001
Alcohol related disorders	1.19 (0.58, 2.46)	0.633	0.91 (0.41, 2.00)	0.806
Cerebrovascular diseases	0.91 (0.62, 1.33)	0.623	0.72 (0.48, 1.08)	0.110
COPD	2.25 (1.53, 3.32)	<0.001	1.84 (1.17, 2.90)	0.008
Malnutrition	2.38 (1.32, 4.28)	0.004	1.86 (0.96, 3.60)	0.066
Heart failure	2.95 (2.16, 4.04)	<0.001	1.97 (1.35, 2.89)	<0.001
Diseases of liver	1.80 (1.13, 2.86)	0.014	1.62 (0.98, 2.67)	0.061
COVID-19	0.25 (0.04, 1.81)	0.171	0.63 (0.20, 1.99)	0.430

COPD: chronic obstructive pulmonary disease; OR: odds ratio; CI: confidence interval.

### 3.6 Association between VDD and 1–12 m outcomes

To assess longer-term outcomes, we examined the association between VDD and adverse events during the 1–12 month period following stroke (Table 6). After propensity score matching (*n* = 4,550 per group), VDD remained significantly associated with increased risks of AKI (HR = 1.32, 95% CI: 1.13–1.55, *p* = 0.001) and all-cause mortality (HR = 1.60, 95% CI: 1.32–1.95, *p* < 0.001) during this extended follow-up period. Furthermore, VDD was associated with elevated risks of ICU admission (HR = 1.37, 95% CI: 1.11–1.69, *p* = 0.003), pneumonia (HR = 1.36, 95% CI: 1.10–1.69, *p* = 0.005), and progression to end-stage renal disease (HR = 1.69, 95% CI: 1.16–2.46, *p* = 0.006).

**Table 6:** Association between vitamin D deficiency (VDD) and 1–12 m outcomes.

Outcomes	VDD group (*n* = 4,550)	Control group (*n* = 4,550)	HR (95% CI)	*p*-value
	Events (%)	Events (%)		
Acute kidney injury	337 (7.4%)	277 (6.1%)	1.32 (1.13–1.55)	0.001
All-cause mortality	251 (5.5%)	169 (3.7%)	1.60 (1.32–1.95)	<0.001
ICU admission	199 (4.4%)	156 (3.4%)	1.37 (1.11–1.69)	0.003
Pneumonia	183 (4.0%)	145 (3.2%)	1.36 (1.10–1.69)	0.005
ESRD	69 (1.5%)	44 (1.0%)	1.69 (1.16–2.46)	0.006

ICU: intensive care unit; ESRD: End-Stage Renal Disease; HR: Hazard ratio; CI: confidence interval.

## 4 Discussion

Our results demonstrated that VDD was significantly associated with increased risk of AKI following ischemic stroke, with VDD patients showing a 55% higher likelihood of developing AKI within 30 days compared to those with sufficient vitamin D levels. This association remained robust across multiple sensitivity analyses. Additionally, patients with VDD exhibited significantly higher risks of mortality, ICU admission, pneumonia, and dialysis requirement within 30 days post-stroke. Vitamin D insufficiency (20–29 ng/mL) was also associated with increased AKI risk, but to a lesser extent than deficiency, suggesting a dose-dependent relationship. The long-term follow-up revealed that VDD’s adverse impact persisted beyond the immediate post-stroke period, with significantly higher risks of AKI, mortality, ICU admission, pneumonia, and progression to end-stage renal disease during the 1–12 month period, indicating the enduring consequences of pre-stroke vitamin D status.

In the current study, pre-stroke VDD was associated with a significantly higher likelihood of developing AKI following ischemic stroke, and this association remained robust across all sensitivity analyses. These adverse renal effects persist long-term, with vitamin D deficient patients showing elevated risks of AKI and progression to end-stage renal disease during the 1–12 month follow-up period. To our knowledge, this is the first study to investigate the relationship between pre-stroke vitamin D status and post-stroke AKI risk. Our findings align with those of Braun et al. (21), who reported a similar adjusted OR of 1.50 for AKI risk in critically ill adults with VDD compared to our observed OR of 1.55 in post-stroke patients. However, our study uniquely demonstrates this relationship specifically in the post-stroke setting. The biological plausibility is supported by experimental evidence of vitamin D’s renoprotective effects through anti-inflammatory, antioxidant, and anti-fibrotic pathways (35–37).

The results of current study suggest that incorporating vitamin D status assessment into risk stratification protocols may be beneficial, particularly for patients with established AKI risk factors. The observed dose-dependent relationship indicates that even modest improvements in vitamin D status might yield renoprotective benefits. While these findings suggest vitamin D supplementation as a potential preventive intervention, this hypothesis requires validation through rigorous randomized controlled trials. Accordingly, we emphasize that our results do not justify immediate clinical implementation of vitamin D supplementation for stroke patients; rather, they underscore a hypothesis-generating association that warrants confirmation in prospective interventional studies. Future research should elucidate the optimal therapeutic regimen including timing, dosage, and duration of supplementation, as well as investigate the underlying pathophysiological mechanisms through which VDD predisposes to post-stroke AKI, potentially informing targeted preventive strategies.

Vitamin D likely confers renoprotective effects in post-stroke patients through several mechanisms (24–29). First, vitamin D regulates the renin-angiotensin-aldosterone system (RAAS) (24, 25), with deficiency leading to RAAS hyperactivation that promotes vasoconstriction, sodium retention, and reduced renal perfusion—particularly problematic during hemodynamic instability following stroke. Second, vitamin D possesses anti-inflammatory properties, modulating cytokine production and reducing oxidative stress that may otherwise exacerbate renal injury after stroke-induced systemic inflammation (26, 28). Third, vitamin D maintains endothelial function (27) and vascular integrity by upregulating nitric oxide production and reducing endothelin-1, potentially preserving renal microcirculation during post-stroke hypoperfusion states. Finally, vitamin D may mitigate the nephrotoxic effects of medications commonly used in stroke management. These mechanisms likely converge to create a physiological environment where adequate vitamin D levels protect against AKI following ischemic stroke.

Our findings regarding the relationship between vitamin D deficiency and post-stroke mortality align with those reported by Daubail et al. (38) and Wajda et al. (39), who demonstrated that lower 25(OH)D levels were associated with increased long-term mortality following stroke. However, our study uniquely demonstrates that this mortality risk begins with pre-stroke vitamin D status (OR = 1.63), suggesting VDD serves as a pre-stroke risk marker rather than just reflecting acute-phase responses; moreover, we observed a dose-dependent relationship with mortality risk significant only in deficient (<20 ng/mL) but not insufficient (20–29 ng/mL) patients, complementing Wajda’s finding that severe deficiency (<10 ng/mL) was a particularly strong predictor of post-stroke mortality (39).

The association between pre-stroke VDD and increased risk of pneumonia in stroke patients extends the findings of Huang et al. (40), who reported that reduced vitamin D levels measured within 24 h after admission were associated with stroke-associated pneumonia. Our study, however, uniquely demonstrates that this relationship exists with pre-stroke vitamin D status rather than post-stroke levels. Additionally, our substantially larger sample size (*n* = 8,686 matched patients versus 863 patients (40)) provides more robust evidence for this association, while confirming the dose-dependent relationship suggested by previous research (40). This is particularly significant given that pneumonia is a leading cause of mortality in stroke patients, accounting for approximately 30% of post-stroke infections (41). Vitamin D has well-established immunomodulatory effects (42), with deficiency potentially compromising immune responses to respiratory pathogens and increasing susceptibility to post-stroke pneumonia. Beyond its general immunoregulatory role, vitamin D enhances innate immunity through the induction of antimicrobial peptides such as human cathelicidin (43, 44), which exhibits direct bactericidal and antiviral activity within respiratory epithelium. Moreover, vitamin D contributes to maintaining endothelial integrity and reducing vascular permeability, thereby limiting inflammatory cell infiltration and pulmonary edema that predispose to infection (45, 46). These mechanisms collectively help explain the observed association between VDD and higher pneumonia risk among stroke patients.

Several independent risk factors were identified for AKI in vitamin D-deficient stroke patients, with notable differences in risk profiles. The strong gender disparity, with females showing 44% lower risk, aligns with current literature showing males have higher AKI susceptibility (47). This may reflect sex-specific differences in renal hemodynamics, hormonal influences on the renin-angiotensin system, and possibly gender-based variations in vitamin D metabolism (47, 48). Pre-existing chronic kidney disease emerged as a significant risk factor, consistent with the “acute-on-chronic” phenomenon where reduced renal reserve amplifies vulnerability to additional insults. Heart failure substantially increased AKI risk, likely through hemodynamic mechanisms including reduced cardiac output and venous congestion. In current study, the heightened risk of AKI associated with COPD (adjusted OR = 1.84) aligns with recent findings from Kwok et al. (49), who demonstrated that COPD patients with hospitalized acute exacerbations had significantly higher risk of developing AKI (adjusted HR = 2.43). This association likely reflects multiple pathophysiological mechanisms including shared inflammatory pathways, hypoxemia-induced renal vasoconstriction, and potential nephrotoxicity from medications commonly used in COPD management (50, 51). These findings suggest that vitamin D-deficient patients with these comorbidities warrant particularly vigilant monitoring for AKI following stroke, and may represent priority populations for future research on preventive interventions.

Building upon the current findings, several important questions remain to be addressed in future studies. Future research should clarify whether vitamin D deficiency represents a causal factor or merely a surrogate marker of frailty and comorbidity burden. Randomized controlled trials evaluating the impact of vitamin D supplementation before or after stroke on AKI and mortality risk are warranted to test the causal hypothesis. Furthermore, mechanistic studies exploring the interplay between vitamin D, systemic inflammation, and the brain–kidney axis could help elucidate underlying biological pathways. Additional investigations should also assess the influence of stroke severity, acute management strategies, and potential sex- or ethnicity-specific differences on the vitamin D–AKI relationship to refine individualized preventive strategies.

This study has several important limitations that warrant consideration. First, its retrospective observational design precludes establishing causality between VDD and post-stroke outcomes, only allowing for associations to be identified. Second, despite extensive propensity score matching, residual confounding from unmeasured variables cannot be ruled out, potentially influencing the observed relationships. Third, the TriNetX database does not provide complete information on stroke severity indicators such as the National Institutes of Health Stroke Scale (NIHSS). As stroke severity is a key determinant of both outcomes and complications, the absence of this variable may lead to residual confounding despite propensity score matching. Moreover, the database structure only permitted matching on pre-stroke comorbidities and treatments, not on acute stroke therapies, which may substantially influence outcomes. Fourth, the vitamin D measurements were obtained within 6 weeks pre-stroke, which may not perfectly represent patients’ long-term vitamin D status or account for seasonal variations. Further, the absence of information on vitamin D supplementation before or after stroke and seasonal variability might have influenced outcomes. These limitations may have led to exposure misclassification, thereby affecting the accuracy of vitamin D status assessment. Consequently, the observed associations should be interpreted with caution, as the true long-term exposure could differ from the measured values. Fifth, because the TriNetX platform does not provide serum creatinine trajectories or urine output data, the standardized KDIGO diagnostic criteria for AKI could not be applied. This reliance on administrative codes may have introduced misclassification bias, potentially affecting the reported incidence rates and attenuating the observed associations. Additionally, we lacked data on the timing and causes of AKI relative to stroke onset, limiting our understanding of the mechanistic relationship. Finally, because most data originated from U.S. healthcare organizations, the findings may not be generalizable to populations with different ethnic backgrounds or healthcare systems. Differences in vitamin D metabolism, sun exposure, supplementation practices, dietary patterns, and stroke management protocols across regions may influence both serum vitamin D status and clinical outcomes. These geographic and ethnic variations should be considered when extrapolating our results to non-U.S. populations, as they may limit the external validity of our findings.

## 5 Conclusion

Our findings demonstrate that pre-stroke VDD was associated with an increased risk of AKI and other adverse outcomes following ischemic stroke, both in short-term and long-term follow-up periods. The observed dose-dependent relationship between vitamin D levels and outcomes suggests potential biological plausibility. Given the significant impact of AKI on stroke recovery and long-term prognosis, our results highlight the importance of recognizing pre-stroke vitamin D status as a modifiable risk factor. Future research should include prospective studies to confirm our findings, randomized controlled trials to evaluate whether vitamin D supplementation before or after stroke reduces AKI incidence and improves outcomes, investigation of underlying pathophysiological mechanisms connecting VDD to post-stroke AKI, and development of personalized risk stratification tools incorporating vitamin D status to identify high-risk patients who might benefit from targeted preventive interventions.

## Data Availability

The raw data supporting the conclusions of this article will be made available by the authors, without undue reservation.
